# Circularly Polarized Luminescence in Composite Films: A Combination of Perovskites and Chiral Nematic Liquid Crystals

**DOI:** 10.3390/molecules29225347

**Published:** 2024-11-13

**Authors:** Guang Chen, Lingtong Meng, Shuting Liu, Liang Peng

**Affiliations:** School of Information and Electrical Engineering, Hangzhou City University, Hangzhou 310015, China; 18906432896@163.com (L.M.); liust@hzcu.edu.cn (S.L.); pengl@hzcu.edu.cn (L.P.)

**Keywords:** circularly polarized luminescence, perovskites, chiral nematic liquid crystals

## Abstract

Chiral inorganic nanomaterial-based circularly polarized luminescence (CPL) materials have shown substantial promise in multiple research areas. However, the luminescence dissymmetry factor (*g*_lum_), a key parameter for CPL, is far from satisfactory, especially for inorganic molecules with high luminescent quantum efficiency and diverse shapes and sizes. Obtaining large *g*_lum_ values is an urgent and crucial task in the field of CPL research. Among different approaches, the combination of inorganic nanomaterials and chiral nematic liquid crystals (N*-LCs) offers distinct advantages in achieving high *g*_lum_ values due to their distinctive optical characteristics and remarkable versatility. This concise review systematically investigates the recent advancements in CPL-active materials consisting of perovskites and N*-LCs. It elaborates on their preparation techniques, optical characteristics, and potential applications. Additionally, a brief outlook on their future development is offered. It is expected that this combination will assume an increasingly significant role in the CPL research field and attract more researchers to explore this area.

## 1. Introduction

Chirality is ubiquitous across all length scales, from subatomic particles to galaxies, signifying an asymmetric configurational property where an object and its mirror image are non-superimposable. Based on the interaction between chirality and electromagnetic waves, various optical effects have emerged, among which circular dichroism (CD) and circularly polarized luminescence (CPL) are particularly prominent [1,2,3]. CPL, which involves the differential emission intensity of left- and right-handed circularly polarized light, can reflect the structural information of chiral luminescent materials or systems in the excited state. Recently, the field of chiroptical materials with CPL has attracted substantial attention because of their potential applications in 3D imaging [4], optical data storage [5,6,7], CPL lasers [8], optoelectronic devices [9,10,11], and chemical sensors [12]. Chiral luminescent systems generate CPL, which is characterized by the distinct emission intensities of left- and right-handed circularly polarized light. By examining CPL behavior, one can obtain the structural information of chiral luminescent systems in the excited state, and this can be utilized to detect the generation, transfer, and amplification of excited-state chirality. The luminescence dissymmetry factor (*g*_lum_), which is used to quantify the degree of CPL, is defined as *g*_lum_ = 2[*I*_L_ − *I*_R_]/[*I*_L_ + *I*_R_], where *I*_L_ and *I*_R_ are the intensities of left-handed and right-handed circularly polarized light, respectively [13]. It is evident that ±2 represents pure CPL emission, with +2 corresponding to completely left-handed CPL and −2 to purely right-handed CPL. Up to now, considerable efforts have been dedicated to understanding CPL materials, such as chiral transition metal complexes, chiral conjugated polymers, chiral organic small molecules, chiral luminescent lanthanide complexes, chiral supramolecular systems, and chiral liquid crystals. For practical applications, there is an urgent need to develop an appropriate CPL material with a high degree of polarization. Most CPL materials find it challenging to provide high asymmetry factors that can meet the requirements of certain optoelectronic applications. Recently, chiral nematic liquid crystals (N*-LCs), which are self-organized helical superstructures, have received more attention. Since the self-organized helical superstructures of N*-LCs have many unique optical properties, such as selective reflection, circular dichroism, and optical rotation, it is expected that N*-LCs will provide new opportunities for the development of CPL-active materials with excellent properties [5,14,15,16,17,18].

CPL-active inorganic nanostructures, which occupy an intermediate size range between angstrom-scale molecules and micrometer-scale objects, offer opportunities for controlling the chirality of light and the reactions of asymmetric synthesis [19,20]. The discovery of CPL in inorganic nanostructures, including chiral II-VI group semiconductor nanostructures, perovskites, metal nanocrystals, transition metal dichalcogenides, and others, has spurred the rapid progress of fundamental and applied research in biology, physics, chemistry, and medicine [21,22,23,24,25]. Perovskite quantum dots, having the general formula ABX_3_ (where A denotes a monovalent cation, B is a divalent metal, and X is a halogen), have emerged as an intriguing semiconductor nanomaterial due to their outstanding properties like bright and narrow-band emission, high photoluminescence, precisely tunable band gaps, and solution processability [26,27,28,29,30]. Since most chiral perovskites originating from chiral inducers possess relatively small *g*_lum_ values, typically in the range of 10⁻^5^ to 10⁻^2^ [5,31], the template-directed self-assembly method capable of generating ordered chiral nanostructures has been considered a highly promising platform for amplifying *g*_lum_.

N*-LCs come in diverse forms, with cholesteric liquid crystals (CLCs) being a notable example. At the molecular level, CLCs are chiral, and on the macroscopic scale, they present non-superimposable helical superstructures. The helical molecular configuration is characterized by two crucial parameters: twist-handedness and helical pitch (P). Specifically, P indicates the distance over which the director of the CLC makes a full 360-degree rotation. According to Bragg’s law, CLCs show selective reflection of CP light. At normal incidence, the maximum wavelength of the reflected light is given by *λ* = n*P*, where n is the average refractive index of the LCs. In materials constructed using CLCs for CPL, the CPL performance is closely related to the optical properties of CLCs. There are two possible ways in which the *g*_lum_ can be amplified in CLCs. In the first mechanism, the optical rotation from the helical superstructure of the CLCs results in a high degree of CPL polarization when the emission peak does not coincide with the reflection band. Hence, the handedness of the CLCs mainly determines the polarized direction of the obtained CPL. The second mechanism is based on CD: when the emission peak matches the reflection band, CPL with the same sense as the helical twist of the helical superstructure is completely reflected, while CPL with the opposite handedness is transmitted. As a result, an extremely high *g*_lum_ can be obtained in this situation [32,33,34].

N*-LCs possessing long-range ordered self-organized chiral superstructures have recently received significant recognition as soft templates for chiral perovskites. This is because they can adaptively guide the assembly of nanosized luminescent building blocks into highly ordered chiral nanomaterials on a larger scale. Furthermore, a very large *g*_lum_ can be achieved when the emission peaks are situated within the photonic band gap (PBG) of N*-LCs, owing to the selective Bragg reflection effect. To date, three methods have been reported for obtaining N*-LCs-directed perovskites. The first is to directly stack an N*-LCs layer that acts as a circular polarizer on an achiral perovskite coating [35]. The second is to use nanoporous and polymeric N*-LCs films as chiral templates and introduce originally nonchiral perovskite crystals into the nanopores [36]. The third is to directly physically blend nonchiral perovskite into a low-molecular-weight N*-LCs matrix [37]. In this part, we highlight the recent advancements in perovskite nanomaterials related to N*-LCs, as well as the *g*_lum_ of chiral inorganic systems originating from the three possible mechanisms mentioned earlier. We will explore the synthesis approaches of these materials, their design principles, and their *g*_lum_ values. Furthermore, we will present the remaining challenges and future research perspectives in this field, both in terms of ongoing basic research and potential applications. This may offer timely guidance for researchers to design CPL-active inorganic nanomaterials with high dissymmetry factors.

## 2. N*-LCs on Achiral Perovskite as Circular Polarizer

Metal halide perovskites, as newly emerged light-emitting materials, have the benefits of high photoluminescence yield, a narrow emission band, and an emission wavelength that can be widely tuned. These advantages have spurred a great deal of research on perovskite-based light-emitting technologies, including light-emitting diodes, lasers, and CPL. The perovskite nanocrystals incorporated into the N*-LCs matrix exhibit substantial polar–environmental stability and possess a high *g*_lum_ value, resulting in an incomparably stable CPL.

**Figure 1:**
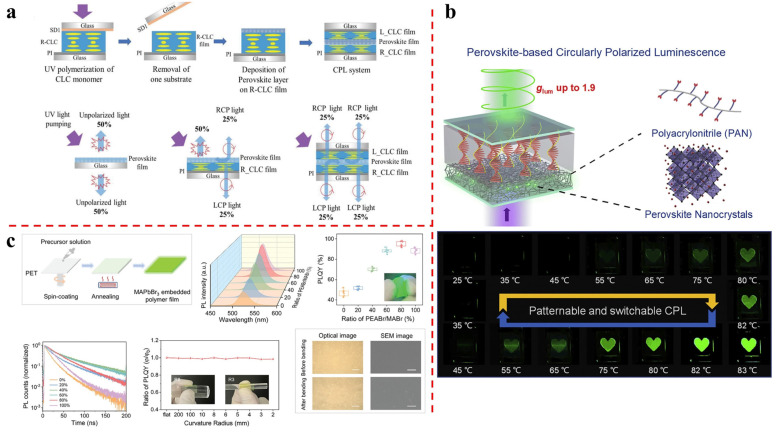
(**a**) The process of creating the cholesteric superstructure stack and the principle underling the operation of the CPL system. Reproduced with permission: Copyright 2019, Wiley−VCH [38]. (**b**) Through the embedding of perovskites into a polymer matrix and the coupling with stimuli−responsive soft helix to build bilayer structured devices, robust CPL with dissymmetry factors reaching up to 1.9 and extraordinary stability has been demonstrated. Reproduced with permission: Copyright 2022, Elsevier [35]. (**c**) The schematic representation of the manufacturing process of perovskite−embedded polymer films on a flexible substrate. Reproduced with permission: Copyright 2024, Wiley−VCH [39].

Li’s group presents a novel method for attaining circularly polarized light emission [38]. This method employs a simple structure in which inorganic perovskite nanocrystals are embedded within a cholesteric superstructure stack with a predefined handedness. The helical-structured cholesteric liquid crystal film functions as a selective filter, transforming the unpolarized light emission from perovskite nanocrystals into CPL. This circularly polarized luminescence has a high dissymmetry factor of up to 1.6, a well-defined handedness, a high photoluminescence quantum yield, and the availability of full color. The CPL is angular-dependent and can be adjusted by shifting the overlap between the reflection band and the emission band. Compared to previous methods, the proposed approach is more straightforward and effective, creating new opportunities for applications in fields such as 3D displays, spintronics, and quantum computation within the scope of optoelectronic and photonic devices (Figure 1a).

Zhu’s group reports the achievement of stable CPL with a dissymmetry factor up to 1.9 by embedding perovskite nanocrystals into a polymer matrix and building a bilayer-structured device with chiral liquid crystals [35]. Polyacrylonitrile is determined as the optimal polymer matrix, not only improving the luminescent efficiency of perovskites to near-unity but also significantly enhancing their environmental stability. The bilayer devices enable graphical patterning, wavelength-tunable, and thermally reversible-switchable CPL. They exhibit great potential for full-color CPL display and anti-counterfeiting applications, successfully addressing major challenges in CPL practical implementation and making significant progress in the development of CPL materials and devices (Figure 1b). Furthermore, his group presents a flexible CPL-active composite film combining a liquid crystal polymer (LCP) film and a perovskite nanocrystalline embedded polymer film. Phenylethylammonium bromide is used as a passivator to enhance the photoluminescence quantum yield of perovskite-embedded polyacrylonitrile films, achieving 98.3% with an optimal ratio [39]. The resulting perovskite–LCP composite film shows a high *g*_lum_ value of −1.81 (highest for flexible CPL films) by using ITO-coated glass for LCP film fabrication. It has excellent mechanical and environmental stabilities, with negligible *g*_lum_ degradation after 1000 bending cycles and stability over one year. Patterned perovskite–LCP composite films are designed for anti-counterfeiting applications, with the ability to show pattern variations and information encryption effects, demonstrating their potential as novel anti-counterfeiting methods and flexible labels for curved surfaces (Figure 1c).

## 3. Nanoporous N*-LCs Film as Chiral Templates for Perovskite Introduction

Chiral perovskites, with their unique chiroptical properties and potential applications in various fields, have become a focus of significant research interest. However, the existing fabrication techniques typically involve intricate chemical synthesis pathways, which may pose challenges in terms of process complexity and cost. In light of this, a novel approach is proposed that utilizes nano-templates made up of cholesteric polymeric networks to introduce chirality into nonchiral perovskites. This alternative method offers a potentially more straightforward and controllable way to achieve chiral perovskites, opening up new possibilities for their further development and application.

**Figure 2:**
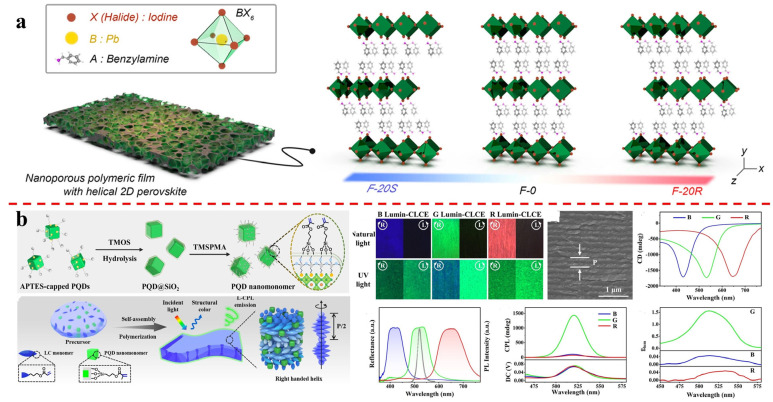
(**a**) An alternative method is proposed to introduce chirality into nonchiral hybrid organic−inorganic perovskites using nano−templates consisting of cholesteric polymeric networks. Reproduced with permission: Copyright 2024, American Chemical Society [36]. (**b**) Fabrication and characterization of Lumin−CLCE films possessing right−handed helicoidal nanostructures. Reproduced with permission: Copyright 2024, Wiley−VCH [40].

Choi’s group proposes a simple method to introduce chirality into nonchiral perovskites using cholesteric polymer network nano-templates [36]. Hybrid organic–inorganic perovskites (HOIP) precursors are incorporated into porous cholesteric polymer templates, and two-dimensional perovskites grow in nanopores. Circularly polarized light emission is observed, and its mechanism depends on the relationship between the cholesteric band gap and the perovskite PL band. When the cholesteric periodicity overlaps with the two-dimensional HOIP emission range, the selective reflection effect is prominent. When it deviates, circularly polarized light emission is due to induced chirality. Circular dichroism analyses support the chiral templating effect. The two-dimensional HOIP in the template has a long exciton lifetime and good stability. This method facilitates the fabrication of two-dimensional chiral HOIPs without complex chemical synthesis steps, showing potential for producing high-performance optoelectronic materials with chiral perovskites (Figure 2a).

Feng’s group reports the design and synthesis of luminescent cholesteric liquid crystal elastomer (Lumin-CLCE) films [40]. Polymerizable perovskite quantum dot nanomonomers with single core–shell structures were designed and synthesized to improve perovskite quantum dots’ stability and compatibility. Lumin-CLCE films with circularly polarized structural color and CPL were fabricated via liquid crystal-templated chiral self-assembly and in situ photopolymerization. The films showed excellent chiroptical properties. CPL intensity was regulated by PBG and fluorescence emission peak positions. The temperature of the UV photopolymerization reaction affected CPL intensity. Biaxial stretching achieved mechanically tunable CPL switching, while uniaxial stretching did not. Lumin-CLCE films were applied to dynamic information encryption and decryption. This work presents a strategy for fabricating chiral perovskite films with tunable CPL and paves the way for advanced flexible chiral perovskite materials in various applications (Figure 2b).

## 4. Blending Nonchiral Perovskite with Low-Molecular-Weight N*-LCs Matrix

Generally, doping chiral compounds into an achiral nematic liquid crystal has been the favored method for obtaining N*-LCs. By altering the ratio of chiral dopants, the photonic bandgap of N*-LCs can be adjusted with flexibility. When nonchiral perovskite is used as a chromophore, directly physically blending it into a low molecular weight N*-LCs matrix is a straightforward process. This method can also lead to achieving a high *g*_lum_. This is because when the emission of the nonchiral perovskite is located within the photonic bandgap of the N*-LCs matrix, it benefits from the unique optical properties of the N*-LCs, such as selective Bragg reflection. This interaction can enhance the CPL properties and result in a relatively large *g*_lum_ value.

**Figure 3:**
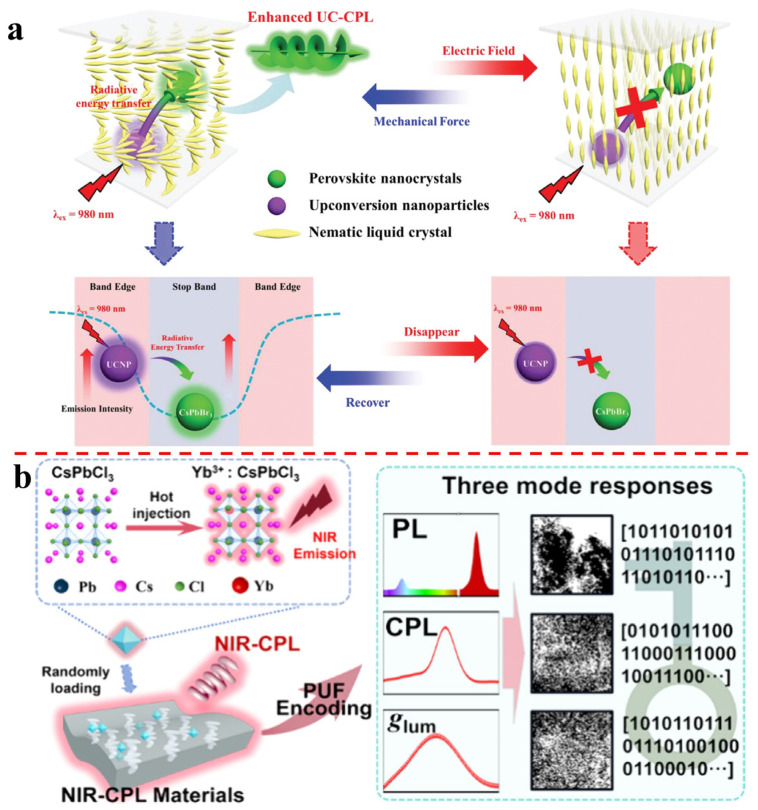
(**a**) A proposed alternative approach is to introduce chirality into nonchiral hybrid organic–inorganic perovskites by using nano-templates made up of cholesteric polymeric networks. Reproduced with permission: Copyright 2020, Wiley-VCH [37]. (**b**) The schematic illustration of the fabrication and response of efficient NIR-CPL physical unclonable functions. Reproduced with permission: Copyright 2024, American Chemical Society [41].

Duan’s group reports enhanced upconverted circularly polarized luminescence (UC-CPL) in a chiral nematic liquid crystal system [37]. By incorporating upconversion nanoparticles (UCNPs) and CsPbBr_3_ perovskite nanocrystals (PKNCs) into NLC, UC-CPL was achieved. The emission spectra of CsPbBr_3_ PKNCs and UCNPs were located at the center and edge of the photonic bandgap of N*-LCs, respectively. The band edge enhancement effect enhanced UCNP emission, which was reabsorbed by CsPbBr_3_ PKNCs. The UC-CPL emission could be switched off by an electric field and recovered by mechanical force. The maximum *g*_lum_ value of UC-CPL reached 1.1. This work provides a strategy for obtaining UC-CPL and shows the first example of achieving a high dissymmetry factor of UC-CPL by steering the photonic bandgap. It has potential applications in various fields, such as chiral optoelectronics and bioimaging (Figure 3a). Furthermore, his group presents optical, physical, and unclonable functions based on near-infrared circularly polarized luminescence (NIR-CPL) [41]. A chiral imprinted photonic (CIP) film with chiral imprinted nanocavities and ytterbium-doped PeNCs (Yb^3^⁺:CsPbCl_3_) was developed. Yb^3^⁺:CsPbCl_3_ was synthesized and characterized, showing efficient chiral imprinted photonic near-infrared (NIR) luminescence. The CIP film was fabricated and characterized, retaining the PBG characteristics. NIR Yb-PeNCs were loaded onto the CIP film, resulting in a NIR-CPL material with a high |*g*_lum_| value. The CIP@Yb-PeNCs film was used to create unclonable labels, and its randomness, uniqueness, and reproducibility were analyzed. The work provides a strategy for integrating NIR-CPL materials with optical, physical, and unclonable functions, enhancing security. The encoding capacity of the NIR-CPL physical unclonable functions materials is increased due to the chiral characteristics and visible light emission. This approach has potential applications in anti-counterfeiting and information security (Figure 3b).

## 5. Conclusions and Outlook

Circularly polarized luminescence materials based on chiral inorganic nanomaterials, particularly those composed of perovskites and chiral nematic liquid crystals, have shown great potential across diverse fields. This review has detailed their recent progress, and the future holds even more promise. Enhancing the luminescence dissymmetry factor is a key priority. Fine-tuning the synthesis and assembly of perovskites and N*-LCs is crucial. Exploring new chiral inducers and optimizing their interaction can lead to more efficient circular polarization and higher *g*_lum_. For instance, using specific ligands or surface modifications to strengthen the chiral coupling could be a viable approach. Improving material stability is essential. This includes enhancing thermal, environmental, and mechanical stability. Developing robust encapsulation methods and modifying the composition can help. For example, advanced encapsulation materials that protect while allowing efficient light emission could be employed. Incorporating additives to enhance stability without compromising optical properties is also worth exploring. Tailoring optical properties is important. Fine-tuning the emission wavelength, bandwidth, and photoluminescence quantum yield can better adapt the materials to various applications. In displays, achieving better color purity, and in sensors, enhancing sensitivity and selectivity are goals. Novel synthesis techniques need exploration. Advanced chemical vapor deposition or solution-based methods with better control over growth and morphology can lead to improved CPL properties. Hybrid fabrication approaches, like combining with other nanostructures or polymers and developing scalable production methods, are vital for practical application, as shown in Table 1.

In application expansion, these materials hold tremendous potential in various areas. In flexible electronics and wearable devices, they can be integrated into flexible displays to offer high-resolution and vivid images with enhanced color purity. The materials can also be utilized in sensors for detecting various physical and chemical parameters, providing real-time data for health monitoring and environmental sensing. As light sources in wearable devices, they can offer energy-efficient and long-lasting illumination. In the biomedical field, these materials can play a crucial role in bioimaging. They can be designed to target specific cells or tissues, providing clear and detailed images for disease diagnosis and treatment monitoring. In drug delivery, they can be used to track the location and release of drugs, ensuring targeted delivery and reducing side effects. For biosensing, they can detect specific biomarkers with high sensitivity and selectivity, enabling early disease detection. In quantum information and optoelectronics, these materials can contribute to quantum communication by providing stable and efficient sources of circularly polarized light. They can also improve the performance of optoelectronic devices such as photodetectors and solar cells. For example, in LED displays, they can enhance color purity and energy efficiency by emitting highly polarized light.

Overall, the combination of perovskites and N*-LCs offers a rich platform for innovation. With continued research, we can expect more advanced materials and applications that will impact optics, electronics, biomedicine, and information technology, driving technological progress and improving our lives. The future looks bright for this area of research, with the potential to open up new possibilities and solutions in various fields. We anticipate seeing significant advancements in the coming years as researchers delve deeper into understanding and exploiting the unique properties of these materials. This will likely lead to the development of more efficient and functional CPL materials and devices, ultimately benefiting a wide range of industries and applications.

## Figures and Tables

**Table 1:** Three methods have been reported for obtaining perovskites directed by N*-LCs.

Method	Liquid Crystal Material	Perovskite	*g*_lum_	Ref.
N*-LCs on achiral perovskite as circular polarizer	Liquid crystal monomer (RM-002), chiral dopant (S1011), and photoinitiator (DMOAP).	CsPbBr_3_, CsPbI_3_ NCs and CsPbBr_3_ NRs	1.6	[38]
	SLC1717 with chiral dopant R-5011 or S-5011.	MAPbBr_3_	1.9	[35]
	Chiral liquid crystal mixture was fabricated by adding Irgacure 184 and chiral dopants S(R)-5011 into the polymerizable LC monomer LC242: RM257.	MAPbBr_3_	1.81	[39]
Nanoporous N*-LCs film as chiral templates for perovskite introduction	LC compound (E7), a chiral dopant (R-811 or S-811), and photoreactive mesogen (RM-242)	2D HOIP (BZA_2_PbI_4_)	0.4	[36]
	RM257, non-reactive mesogen HTG135200 and Irg651	CsPbX_3_ (X = Cl, Br, I) PQD nanomonomers	1.5	[40]
Blending nonchiral perovskite with low-molecular-weight N*-LCs matrix	SLC1717 and R(S)811	CsPbBr3	1.1	[37]
	Polymer precursor C6M, crosslinking agent TMPTA, photoinitiator I-651 and chiral dopant S(R)5011	Ytterbium-doped PeNCs (Yb^3+^:CsPbCl_3_)	0.9	[41]
